# MiRNA-10a is upregulated in NSCLC and may promote cancer by targeting PTEN

**DOI:** 10.18632/oncotarget.4972

**Published:** 2015-07-22

**Authors:** Tao Yu, Lei Liu, Jing Li, Mingxia Yan, Hechun Lin, Ying Liu, Dandan Chu, Hong Tu, Aiqin Gu, Ming Yao

**Affiliations:** ^1^ State Key Laboratory of Oncogenes and Related Genes, Shanghai Cancer Institute, Renji Hospital, Shanghai Jiao Tong University School of Medicine, Shanghai, China; ^2^ Shanghai Chest Hospital, Shanghai Jiao Tong University School of Medicine, Shanghai, China

**Keywords:** NSCLC, metastasis, microRNA-10a, PTEN

## Abstract

MicroRNAs (miRNAs) are involved in human cancer including non-small cell lung cancer (NSCLC). In this study, we compared miRNA expression microarray of SPC-A-1sci (high metastatic) and SPC-A-1 (weakly metastatic) cells. We found that miRNA-10a was up-regulated in NSCLC compared with corresponding normal tissues. High expression of miR-10a was associated with tumor node metastasis and lymph node metastasis. Furthermore, overexpression of miR-10a promoted NSCLC cell proliferation, migration and invasion *in vitro*. We found that PTEN was a direct target of miR-10a in NSCLC. Also miR-10a activated the PTEN/AKT/ERK pathway. We suggest that miR-10a contributes to NSCLC by targeting PTEN.

## INTRODUCTION

Lung cancer is a leading cause of tumor-related deaths worldwide [1, 2]. Non-small cell lung cancer (NSCLC) is a predominant type of lung cancer, accounting for about 85% of all lung cancer cases. It has a very high mortality rate and also a low 5-year survival rate of less than 15% after initial diagnosis [3] with metastasis being the primary reason for patient death [4]. Many factors are involved in the complex process of tumorigenesis [5], therefore uncovering the molecular mechanisms of NSCLC may help to identify effective therapies for NSCLC treatment.

MicroRNAs (miRNAs) are small, non-coding, regulatory RNAs that negatively regulate gene expression at the post-transcriptional and/or translational level by binding the 3′-untranslated region (UTR) [6, 7]. It is estimated that approximately one third to one half of human genes are directly regulated by miRNAs with each miRNA predicted to target several hundred transcripts, making miRNAs one of the largest families of gene regulators [8]. Furthermore, it is reported that miRNAs are implicated in the regulation of various cellular processes, including proliferation, metastasis, differentiation and apoptosis [9–15]. They can function either as key oncogenes or tumor suppressors in tumor progression [16, 17]. Previously, we established a highly metastatic cell line, SPC-A-1sci, generated from weakly metastatic SPC-A-1 cells by *in vivo* selection in mouse models [18]. Using miRNA microarray analysis, we looked for metastasis-related miRNAs in these two cell lines. We discovered that low expression of miR-200c, miR-193a-3p and miR-193a-5p influenced the migration and invasion of NSCLC cell lines [19, 20]. We also found that the expression of miR-10a was up-regulated in NSCLC tumor tissues compared to corresponding noncancerous tissues, and its expression was correlated with metastasis and tumor node metastasis in NSCLC tissue. We then showed that miR-10a promoted the migration, invasion and proliferation in NSCLC cell lines. Additionally, we also found that PTEN (phosphatase and tensin homolog), an important tumor suppressor, was the direct target gene of miR-10a. Consistently, the expression of PTEN was negatively correlated with the expression of miR-10a in NSCLC tissues. Therefore, miR-10a could enhance the growth and metastasis of NSCLC by activating the PTEN/AKT/ERK signaling pathway, thus providing a potential molecular therapeutic target for treatment of NSCLC patients.

## RESULTS

### miR-10a is up-regulated in human NSCLC tissues and associated with NSCLC progression

**Figure 1:**
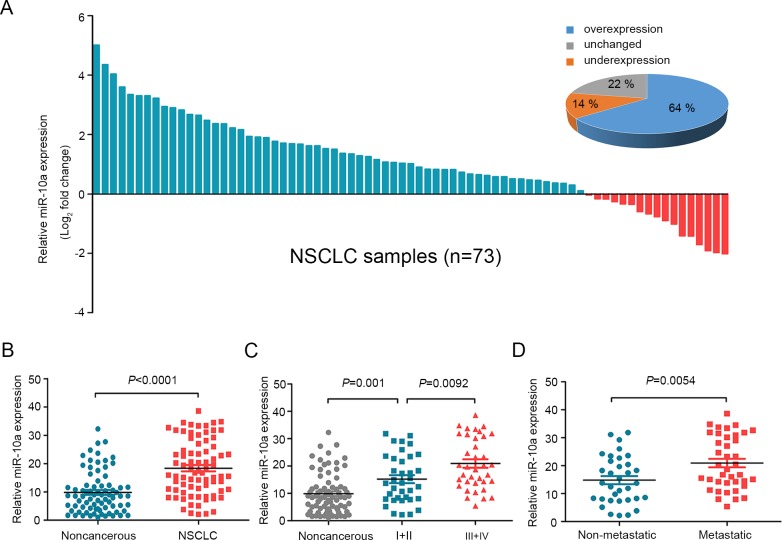
MiR-10a is overexpressed in NSCLC and correlated with clinical stage and tumor metastasis **A.** The expression levels of miR-10a in 73 paired NSCLC and corresponding noncancerous tissues were measured by TaqMan real-time PCR and normalized against an endogenous U6 RNA control. **B.** The expression of miR-10a was overexpressed in NSCLC tissues compared with the noncancerous tissues. **C.** MiR-10a expression was detected in different clinical stages of NSCLC. **D.** The up-regulation of miR-10a in NSCLC was associated with tumor metastasis; the patients were classified into tumor metastasis negative and positive groups (lymph node metastasis and/ or distal metastasis) Error bars represent SEM. The statistical analysis was performed using paired *t* test **B.** and Student's *t* test **C.** and **D.**.

To clarify the biological role of miR-10a in NSCLC cells, we first detected the expression of miR-10a using quantitative RT-PCR (qRT-PCR) in 73 pairs of human NSCLC tissue samples and their corresponding noncancerous lung tissue controls. MiR-10a expression was significantly up-regulated in tumor tissue samples (64%) compared to the controls (Figure 1A and 1B). We then conducted stratified analyses to assess miR-10a expression in NSCLC patients with specific clinical characteristics. As shown in Table 1, there were no differences in miR-10 levels associated with age, gender, tumor size, differentiation and local invasion. However, we found that miR-10a levels were increased in lung cancer with advanced (stage III and IV, *n* = 37) to early stages (stage I and II, *n* = 36) (Figure 1C). Further, miR-10a expression was up-regulated in NSCLC that had lymph node or distal metastasis (*n* = 39) compared with those that had not spread (*n* = 34) (Figure 1D).

**Table 1:** The relationship between miR-10a expression and their Clinicopathologic parameters in 73 of NSCLC Patients

Clinicopathologic parameters	Number of cases	Median expression of miR-10a	
		Mean ± SD	*P*-value
Age			
<60	27	19.056 ± 8.6102	0.6958
≥60	46	18.151 ± 9.9960	
Gender			
Male	45	18.342 ± 9.3248	0.8709
Female	28	18.720 ± 9.8310	
Tumor size(cm)			
≤3	35	18.194 ± 8.9262	0.8025
>3	38	18.754 ± 10.031	
Degree of differentiation			
well and moderately	25	16.910 ± 9.5407	0.3077
poorly	48	19.306 ± 9.4069	
Local invasion			
T1+T2	55	17.544 ± 9.0067	0.1382
T3+T4	18	21.362 ± 10.459	
TNM stage			
Stage I + II	36	15.185 ± 8.8639	0.0092**
Stage III+IV	37	20.905 ± 9.3748	
Metastasis			
No	34	14.826 ± 8.5312	0.0054**
Yes	39	20.925 ± 9.4967	

*P* value represents the probability from a student's test for miR-10a expression between variable subgroups. *P < 0.05, ** *P* < 0.01, which was considered to have a significant difference.

### miR-10a promotes the migration and invasion of NSCLC cells

**Figure 2:**
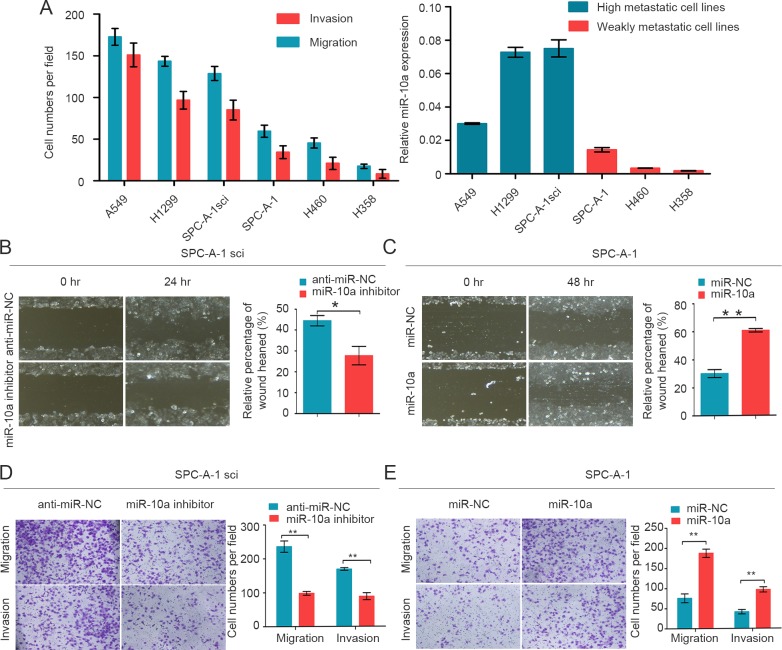
MiR-10a is increased in high metastatic cancer cells and promotes migration and invasion of NSCLC cells **A.** Transwell analysis to determine the migration and invasion of A549, H1299, SPC-A-1sci, SPC-A-1, H460 and H358 cells and miR-10a expression was measured in different NSCLC cells. U6 was used as a control. **B.** and **C.** Would-healing assay for SPC-A-1sci and SPC-A-1 were performed with miR-10a inhibitors, anti-miR-NC (SPC-A-1sci), miR-10a mimics, miR-NC (SPC-A-1). **D.** and **E.** Transwell migration and invasion assays for SPC-A-1sci and SPC-A-1 were determined after transduction with the miR-10a inhibitors, anti-miR-NC, miR-NC and miR-10a mimics. The data are representative of three independent experiments. Error bars represent SD. *P < 0.05; **P < 0.01 by Student's *t* test.

Although miR-10a is strongly conserved across species (Supplementary Figure S1), its role in lung cancer metastasis is unclear. We measured the endogenous expression levels of miR-10a in six lung cancer cell lines (A549, H1299, SPC-A-1sci, SPC-A-1, LC-2 and H358) by using qRT-PCR. The migration and invasion ability of these six human NSCLC cell lines was compared by Trans-well assays (Figure 2A). We found that miR-10a expression was significantly up-regulated in high metastatic lung cancer cells (A549, H1299 and SPC-A-1sci) compared with weakly metastatic lung cancer cells (SPC-A-1, H460 and H358) (Figure 2A).

To verify the effects of miR-10a on lung cancer cell migration and invasion, we transfected SPC-A-1 cells with miR-10a mimics and SPC-A-1sci cells with miR-10a inhibitors. After wound-healing assays, we observed that migration rates were suppressed in SPC-A-1sci cells transfected with miR-10a inhibitors compared to anti-miR-NC (Figure 2B). In contrast, migration rates were enhanced in SPC-A-1 cells transfected with miR-10a mimics compared to miR-NC (Figure 2C). Invasion and migration ability was decreased in SPC-A-1sci cells infected with miR-10a inhibitors compared to control cells (Figure 2D). On the other hand, invasion and migration abilities of SPC-A-1 cells infected with miR-10a mimics were markedly increased compared to control cells (Figure 2E). These results showed that overexpression of miR-10a increased the invasion and migration of human NSCLC cells.

### miR-10a enhances proliferation of NSCLC cells during G2/M phase

**Figure 3:**
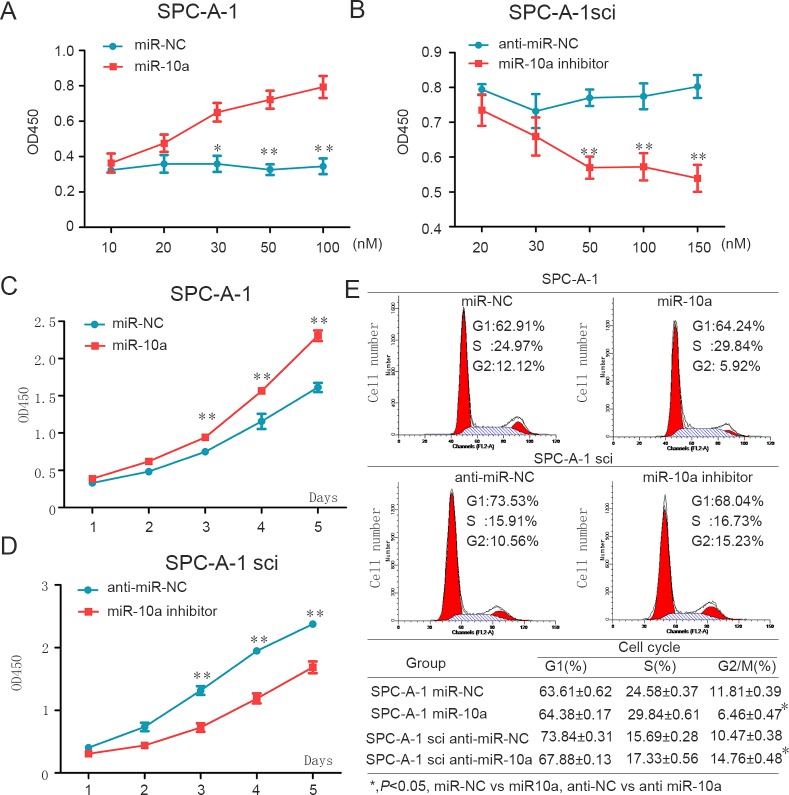
MiR-10a enhances cell proliferation through influencing G2/M cell cycle of NSCLC cells **A.** and **C.** Overexpression of miR-10a promotes SPC-A-1 cell growth. A, different doses of miR-10a mimics or miR-NC was transfected into SPC-A-1 cells. Cell number was counted at 48h after transfection. C, miR-10a mimics or miR-NC was transfected into SPC-A-1 cells. **B.** and **D.** Down-regulation of miR-10a inhibits SPC-A-1sci cell growth. B, different dose of miR-10a inhibitor or anti-miR-NC was transfected into SPC-A-1sci cells. Cell number was counted at 48h after transfection. D, miR-10a inhibitors or anti-miR-NC was transfected into SPC-A-1sci cell. **E.** SPC-A-1 and SPC-A-1sci cell cycle profiles determined by propidium iodide (PI) staining and flow cytometry assays. Percentages of different cell cycle phases were presented. The data are representative of three independent experiments. Error bars represent SD. *P < 0.05; **P < 0.01 by Student's *t* test.

We then analyzed the effect of miR-10a on cell growth in both cell lines, SPC-A-1 and SPC-A-1sci. We found that miR-10a mimics increased proliferation of SPC-A-1 cells in both a dose and time dependent way (Figure 3A and 3C). Consistent with these findings, miR-10a inhibitors suppressed cell growth in SPC-A-1sci cells in both a dose and time dependent way (Figure 3B and 3D).

To gain insight into the function of miR-10a, we examined the effect of miR-10a on cell cycle progression. Compared with miR-NC, miR-10a mimics resulted in a reduced G2/M population in SPC-A-1 cells (Figure 3E). We also confirmed that miR-10a inhibitors enhanced a G2/M population in SPC-A-1sci cells compared with anti-miR-NC (Figure 3E). These findings suggest that miR-10a promoted cell growth during the G2/M phase.

### PTEN is the direct downstream target of miR-10a

**Figure 4:**
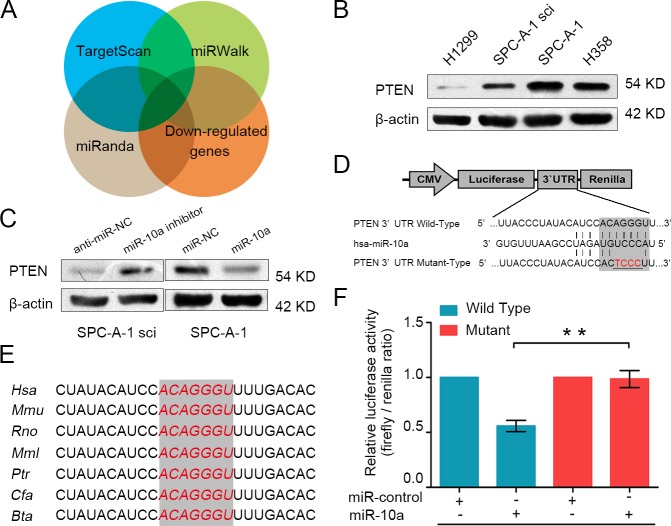
PTEN is a direct target gene of miR-10a **A.** PTEN was identified as potential regulatory target of miR-10a by considering the down-regulation genes from the gene expression profiles and using prediction tools, including TargetScan, miRWalk and miRanda. **B.** The protein expression level of PTEN was measured using Western blot analysis in H1299, SPC-A-1sci, SPC-A-1 and H358. **C.** The protein expression level of PTEN was detected in SPC-A-1sci cells transfected with miR-10a inhibitor or anti-miR-NC and SPC-A-1 cells transfected with miR-10a mimics or miR-NC. **D.** The sequences of the putative miR-10a binding sites in wild type (emphasized with shadow) and mutant (red) PTEN-3′UTR. **E.** The potential binding sequences for miR-10a within the PTEN-3′UTR of human (Hsa), mouse (Mmu), rat (Rno), macaca mulatta (Mml), pan troglodytes (Ptr), dog (Cfa), bos taurus (Bta). **F.** The relative luciferase activity of luciferase reports with wild type or mutant PTEN-3′UTR were determined in HEK 293T cells. Which were co-transfected with the miR-10a mimic or miR-control. Renilla luciferase activity served as an internal control. Statistical significance was observed between the wild type and the mutant groups transfected with miR-10a. The data are representative of three independent experiments. Error bars represent SD. *P < 0.05; **P < 0.01 by Student's *t* test.

In order to explore the mechanism through which miR-10a initiates NSCLC cells progression, we searched for potential regulatory targets of miR-10a using several bioinformatics methods, including TargetScan, miRWalk and miRanda. Moreover, we examined the global mRNA expression profile of SPC-A-1 and SPC-A-1sci cells. We selected potential target genes of miR-10a by using mRNA microarray and prediction tools to identify down-regulated genes in SPC-A-1sci cells (Figure 4A). Of these candidate genes, PTEN is regarded as a tumor suppressor. Tang et al. identified PTEN as the target of Oct4, which suppresses lung cancer progression [21]. Ji et al. found that PTEN is associated with clinicopathologic features of NSCLC [22]. Lang et al. showed that microRNA-429 induces tumorigenesis of NSCLC cells by directly targeting PTEN [23].

To further validate whether PTEN was a target gene for miR-10a, we analyzed the expression of PTEN in highly metastatic (H1299 and SPC-A-1sci) and weakly metastatic (SPC-A-1 and H358) NSCLC cells. As shown in Figure 4B, PTEN was down-regulated in highly metastatic cells. Moreover, we used Western blot analysis to demonstrate that high expression of miR-10a dramatically suppressed the protein level of PTEN, whereas the inhibition of miR-10a significantly enhanced the expression of PTEN (Figure 4C). Analysis of the 3′UTR sequence of PTEN using TargetScan revealed one possible binding site for miR-10a, which is strongly conserved in human, mouse, rat, macaque, chimpanzee, dog and cattle (Figure 4E). To determine whether PTEN is regulated by miR-10a through direct binding to its 3′UTR, we constructed the 3′UTR fragment of PTEN, including the miR-10a binding site (Figure 4D), with the corresponding mutant counterpart inserted downstream of the firefly luciferase reporter gene. For luciferase activity assays, miR-10a reduced the relative luciferase activity of the PTEN-3′UTR binding site, whereas luciferase activity was not significantly changed in the mutant binding site (Figure 4F). These results suggest that miR-10a down-regulated PTEN expression by directly targeting its 3′UTR.

### PTEN critically mediates miR-10a in NSCLC cells

**Figure 5:**
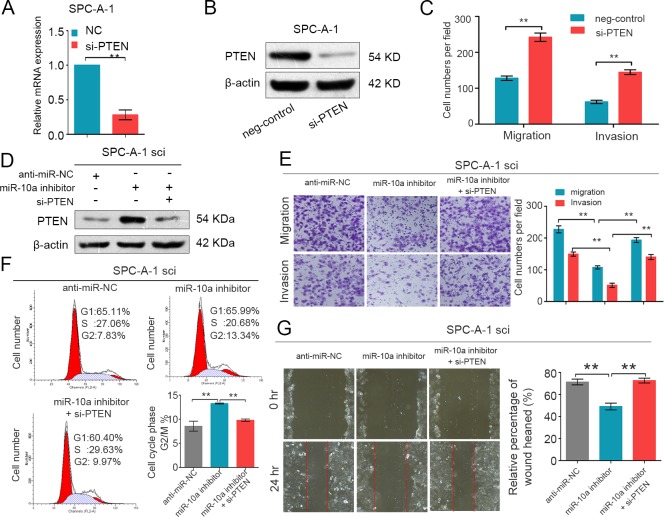
PTEN is involved in the miR-10a-induced promotion of NSCLC cell growth and metastasis (A and B) Real-time PCR and Western blot analyses of PTEN expression in SPC-A-1 cells transfected with si-PTEN or the negative control. β-actin was used as an internal control. **C.** Transwell migration and invasion assays were performed in SPC-A-1sci cells after transfection with negative control (NC) and si-PTEN. **D.** Western blot analysis of PTEN expression in SPC-A-1sci cells after transfection with anti-miR-NC, miR-10a inhibitors and si-PTEN. (E and G)Transwell migration and invasion assays and Would-healing assays for SPC-A-1sci were determined after transduction with the miR-10a inhibitors, anti-miR-NC and si-PTEN. **F.** Representative images and the table depict the results of cell cycle assays in SPC-A-1sci. The data are representative of three independent experiments. Error bars represent SD. *P < 0.05; **P < 0.01 by Student's *t* test.

It has been reported that PTEN is closely associated with lung cancer migration and proliferation [24, 25]. To explore whether PTEN is involved in miR-10a-induced promotion of NSCLC cell migration and proliferation, we knocked down endogenous PTEN expression using specific siRNA in SPC-A-1 cells. As shown in Figure 5A and 5B, si-PTEN significantly reduced the expression of PTEN mRNA and PTEN protein levels. Transwell assays showed that si-PTEN promoted the migration and invasion of SPC-A-1 cells (Figure 5C). Consistently, si-PTEN accelerated the growth and wound healing of SPC-A-1 cells (Supplementary Figure S2).

We further assessed whether miR-10a promoted NSCLC cell growth and metastasis through repression of PTEN expression. We co-transfected both siRNA against PTEN (si-PTEN) and miR-10a inhibitors into SPC-A-1sci cells. We then used Western blot analysis to evaluate the expression of PTEN in these cells (Figure 5D). As expected, knockdown of PTEN expression attenuated the suppression effects of the miR-10a inhibitors on migration and invasion in SPC-A-1sci cells (Figure 5E). Additionally, si-PTEN also mediated miR-10a's ability to promote cell growth and wound healing in NSCLC cells (Figure 5F and 5G). Taken together, we have provided further evidence that PTEN is a direct and functional target gene of miR-10a on NSCLC.

### miR-10a is inversely correlated with PTEN expression in NSCLC tissues

**Figure 6:**
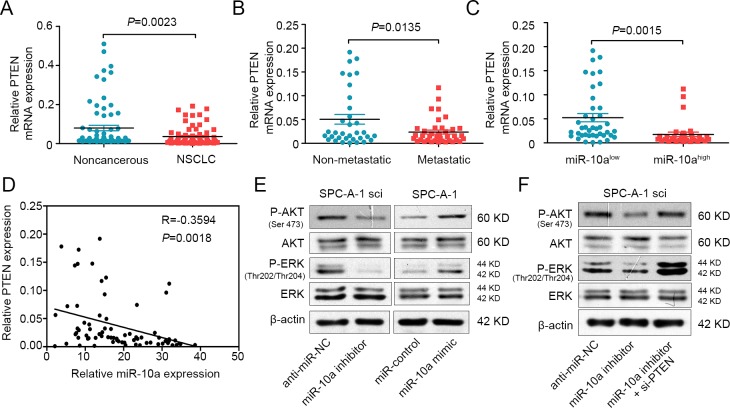
MiR-10a is inversely correlated with PTEN in NSCLC and mediates the activation of AKT and ERK **A.** Real-time PCR analysis to quantify the endogenous expression levels of PTEN in NSCLC patients compared with the noncancerous tissues. **B.** The expression level of PTEN was detected in metastatic NSCLC tissues compared with non-metastatic tissues. **C.** The expression level of PTEN was measured in the presence of low and high miR-10a expression levels. **D.** Paired analyses between miR-10a and PTEN expression in NSCLC tissues. **E.** Western blot analysis of P-AKT and P-ERK in SPC-A-1sci cells transfected with miR-10a inhibitor or anti-miR-NC and SPC-A-1 cells transfected with miR-10a mimics or miR-NC. **F.** Western bolt analysis of P-AKT and P-ERK in SPC-A-1sci cells transfected with anti-miR-NC, miR-10a inhibitor and si-PTEN. The data are representative of three independent experiments.

miR-10a can directly bind the PTEN seed sequence, possibly decreasing the expression of PTEN. To test this, we used qRT-PCR to compare the expression of PTEN in 73 cases of primary lung cancer tissue to the expression in adjacent, non-cancerous tissue. Compared to the adjacent tissues, the expression of PTEN was down-regulated (Figure 6A). We also found that PTEN expression was down-regulated in NSCLC that had lymph node or distal metastasis compared with those had not spread (Figure 6B), and the expression of PTEN was significantly decreased in lung cancer with advanced (stage III and IV, *n* = 37) to early stages (stage I and II, *n* = 36) (Supplementary Figure S3 A and B). Moreover, there was an inverse relationship between miR-10a and PTEN expression in NSCLC tissues (Figure 6C and 6D).

### MiR-10a regulates the PTEN/AKT/ERK signaling pathway

As PTEN can regulate the activity of AKT and ERK pathways [26, 27], we examined whether miR-10a could regulate phosphorylated protein levels downstream of these pathways. As shown in Figure 6E, miR-10a mimic promoted the phosphorylation of AKT (ser473) and ERK (thr202/thr204). Conversely, miR-10a inhibitor suppressed the phosphorylation of AKT and ERK. The total protein levels of AKT and ERK were not changed. Furthermore, we clarified that PTEN could mediate the effect of miR-10a on the phosphorylation of AKT and ERK (Figure 6F). These data indicate that miR-10a promoted the invasion and growth of NSCLC cells by regulating the PTEN/AKT/ERK signaling pathway.

## DISCUSSION

Lung cancer, predominantly by non-small cell lung cancer (NSCLC), is one of the most deadly types of cancer [3, 28]. Although great progress has been made in early diagnosis and treatment methods in recent decades, its poor clinical outcome underscores a compelling need for further understanding of the underlying molecular mechanisms of NSCLC [29]. Recently, numerous evidences indicate that many miRNAs are characterized and play critical roles in cancer progression, suggesting that they could provide new sights into the biological mechanism of this disease. In cancers, miRNA can act on both tumor oncogenes and tumor suppressors to exert a wide range of effects on tumorigenesis [30], proliferation [9], apoptosis [31]and metastasis [32].

In this study, the miRNA expression profiles of SPC-A-1 and SPC-A-1sci cells provide an important method to screen metastasis-associated miRNAs. In the present study, we demonstrated that the expression of miR-10a is significantly up-regulated in NSCLC tissues compared with non-cancerous tissues, especially in higher tumor stage tissues. To date, up-regulation of miR-10a has been found in several different tumor types. Ohuchida K et al. reported that microRNA-10a is overexpressed in human pancreatic cancer and involved in its invasiveness, partially via suppression of the HOXA1 gene [33]. Ovcharenko D et al. recently showed that miR-10a overexpression is associated with NPM1 mutations and MDM4 down-regulation in intermediate-risk acute myeloid leukemia [34]. Hudson et al. reported that overexpression of miR-10a may be important for tumor development in medullary thyroid carcinoma [35]. However, the association of miR-10a with lung cancer metastasis has not yet been reported. Our findings indicate that miR-10a might act as an oncogene in NSCLC. Gain-of-function and loss-of-function assays were performed to demonstrate the effect of miR-10a on NSCLC growth and metastasis. We showed that miR-10a mimic promoted the migration, invasion and proliferation of NSCLC cells. Consistently, miR-10a has been reported to enhance migration, invasion and growth in other cancer cells. Long MJ et al. reported that microRNA-10a promotes cell growth, migration and invasion in human cervical cancer cells by targeting CHIL1 [36]. Yan Y et al. showed that miR-10a controls glioma migration and invasion through regulating epithelial-mesenchymal transition via EphA8 [37]. It has been reported that miR-10a silencing reverses cisplatin resistance in human lung cancer cell lines via the transforming growth factor-β/Smad2/STAT3/STAT5 pathway [38]. Moreover, we demonstrated that PTEN was identified as the direct target of miR-10a by Western blot and dual-luciferase reporter assays. We also found that the target gene could mediate the function of miR-10a in the migration, invasion and growth of NSCLC cells. Interestingly, Zeng T et al. also showed that miR-10a enhanced the metastatic potential of cervical cancer cells by targeting PTEN [39]. In addition, miR-10a and the expression of PTEN were inversely associated in NSCLC tissues, suggesting that the down-regulation of PTEN might at least partially reflect the up-regulation of miR-10a. We also showed that miR-10a could promote the phosphorylation of AKT and ERK. These results suggest that miR-10a influences the NSCLC progression by regulating the PTEN/AKT/ERK signaling pathway.

To conclude, we have shown that miR-10a might promote the migration, invasion and growth of NSCLC cells through direct targeting of the PTEN/AKT/ERK signaling pathway. Importantly, these findings might provide a promising therapeutic target in NSCLC

## MATERIALS AND METHODS

### Cell lines and cell culture

Human NSCLC cell lines, SPC-A-1, SPC-A-1 sci, A549, H1299, H460 and H358 were cultured at 37°C in a humidified air atmosphere containing 5% CO_2_ in DMEM (SPC-A-1, SPC-A-1 sci, A549, H1299, H460 and HEK-293T), RPMI1640 (H358) supplemented with 10% fetal bovine serum (Biowest, South America origin), 100u/ml penicillin (Sigma-Aldrich), and 100ug/ml streptomycin (Sigma-Aldrich).

### Clinical NSCLC tissue samples

The 73 paired patient samples of primary lung cancer tissues and matched adjacent non-cancerous tissues were obtained the Department of Lung Cancer, Shanghai Chest Hospital affiliated to Shanghai Jiao-tong University from 2011 to 2012. Tissue samples acquired from the routine therapeutic surgery of patients who did not receive anti-tumor treatment. Upon resection, Human surgical specimens were immediately frozen in liquid nitrogen and stored at −80°C refrigerator. Informed consent was obtained from all patients and the research was approved by the Ethics Committee of Shanghai Jiao-tong University.

### RNA extraction and quantitative real-time PCR

MicroRNA was extracted from patient tissue samples and cultured cells using mirVana™ miRNA isolation Kit (Ambion). The expression level of mature miRNA was quantified with specific primers and probes using TaqMan miRNA assays (Applied Biosystems) and normalized by U6 small nuclear RNA according to the manufacturer's instructions. Total RNA was extracted from human tissue samples and cultured cells using TRIzol reagent (Invitrogen, CA) according to the manufacturer's protocol and quantified with Nanodrop 2000 (Thermo, Japan). Complementary cDNA synthesis was performed using the PrimeScript^TM^ RT Reagent Kit (TaKaRa, China). Quantitative Real-time polymerase chain reaction was performed using SYBR Green premix Ex Taq (TaKaRa, China). β-actin was the internal control and was analysed using the 2^−ΔΔCt^ method.

### Cell transfection

The miRNA-10a mimics, and miR-control that were used with transient transfection were designed and synthesized by RiboBio (Guangzhou, China). The miRNA-10a inhibitors were synthesized by BioMics (Nantong, China). The small interfering RNAs (siRNA) were synthesized by BioMics. Cells were transfected with miRNA mimic, miRNA inhibitor and siRNA,using lipofectamine 2000 Reagents (Invitrogen, CA) according to the instructions. For RNA extraction, migration, invasion, and Western blot assays, Cells were obtained 48 hours after transfection.

### Wound-healing assays

For cell motility assay, the lung cancer cells were transiently transfected with miR-10a mimics, miR-10a inhibitors, miR-control and anti-control. Cells were then seeded in six-well plates to near confluence. A single scratch wound was carefully created using a 20 μl sterile pipette tip across the confluent cell monolayer, and the cell debris was removed by washing with PBS and incubated with DMEM (1%FBS). The wounded monolayers were then photographed at 0 h, 24 h and 48 h after wounding.

### *In vitro* cell proliferation assays

For cell proliferation assays, cells were seeded into each well of a 96-well plate (2000 per well) and the cell proliferation ability was determined by the Cell Counting Kit-8 (CCK8) Assay Kit (Dojindo Corp, Japan). 10ul of the kit reagent dissolved with 100μl DMEM was added to each well, and 2h later the absorbance was measured at 450 nm to calculate the number of cells.

### *In vitro* migration and invasion assays

Cell migration and invasion assays were performed using a 24-well plate with 8-μm pore size chamber inserts (Corning). For migration assays, 5×10^4^ cells were placed into the upper chamber per well with the non-coated membrane. For invasion assays, 1×10^5^ cells were placed into the upper chamber per well with the Matrigel-coated membrane which was diluted with serum-free culture medium. In both assays, Cells were suspended in 200 μl of DMEM without FBS when they were seeded into the upper chamber. In the lower chamber, 800 μl of DMEM supplemented with 10% FBS was added. After incubation for 16 h at 37°C and 5% CO_2_, the membrane inserts were removed from the plate, and non-invading cells were removed from the upper surface of the membrane. Cells that moved to the bottom surface of the chamber were fixed with 100% methanol for 20 min and stained with 0.1% crystal violet for 30 min. Then, the cells were imaged and counted in at least 10 random fields using a CKX41 inverted microscope (Olympus, Japan). The assays were conducted three independent times.

### Cell cycle

The lung cancer cells were fixed into 70% ethanol at −20°C for 24 hours, stained with 50 μg/mL propidium iodide (PI) (Kaiji, China), and analyzed using a FACSCalibur flow cytometer (BD Bioscience, MA). The results were analyzed using ModFit software (BD Bioscience, USA). Assays were conducted three independent times.

### Dual-luciferase reporter assay

The mixture of 50 ng pluc-3′UTR, 10 ng Renilla and 5 pmol miRNA-10a mimic or negative control were co-transfected into HEK-293T cells according to the recommended instruction by using the Lipofectamine 2000. After 48 h of transfection, Firefly and Renilla luciferase activity was measured by the Dual-Luciferase Reporter Assay System (Promega). The relative firefly luciferase activities were detected by normalizing to Renilla luciferase activities which served as an internal control for transfection efficiency.

### Western blot analysis

Cellular proteins were extracted from cultured cells with a mixture of T-PER Protein Extraction Reagent (Thermo), PhosSTOP (Roche) and Complete Mini (Roche). Protein samples were separated in sodium dodecyl sulfate (SDS)-PAGE and transferred to nitrocellulose filter membranes (Millipore, USA). After blocking in phosphate buffered saline (PBS) / Tween-20 containing 5% nonfat milk, the membranes were incubated with the following primary antibodies: AKT (Cell Signaling Technology), PTEN (Cell Signaling Technology), ERK1/2 (Cell Signaling Technology), phosphor-AKT (ser473)(Cell Signaling Technology), phosphor-ERK1/2(thr202/tyr204)(Cell Signaling Technology). β-Actin(Sigma-Aldrich). HRP-conjugated anti-rabbit IgG (Sigma-Aldrich) were incubated as the secondary antibodies. Subsequent visualization was detected with Super Signal West Femto Maximun Sensitivity Substrate (Thermo, Japan).

### Bioinformatics

MiRNA sequence was based on miRBase (microrna.sanger.ac.uk). Potential miRNA targets were predicted and performed using three publicly available algorithms: TargetScan, PicTar, and miRanda.

### Statistical analysis

All the analyses were performed with the SPSS software (version 19.0). Data were imaged with GraphPad Prism 5 software. Quantitative variables were presented as means and S.E.M unless otherwise noted and analyzed by student's *t* test between two groups (two-tailed; *P* < 0.05 was considered statistically significant).

## SUPPLEMENTARY MATERIAL FIGURES


