# Supplementary material for: MiRNA-10a is upregulated in NSCLC and may promote cancer by targeting PTEN
Source: Oncotarget. 2015 Jul 22;6(30):30239–50. doi: 10.18632/oncotarget.4972 (PMC4745794; doi:10.18632/oncotarget.4972)
Supplement: Supplementary file 1 [file oncotarget-06-30239-s001.pdf]

MiRNA-10a is upregulated in NSCLC and may promote cancer by targeting PTEN

Supplementary Material

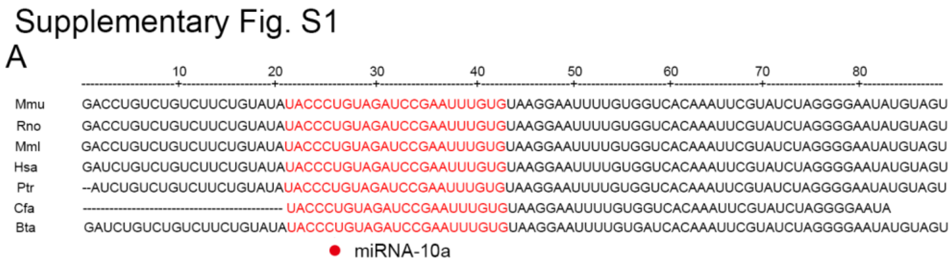

Supplementary Figure S1: sequence alignments of miR-10a from various species obtained from the miRNA database (miRBase v20). Red letters indicate sequences corresponding to miR-10a.

Supplementary Fig. S2

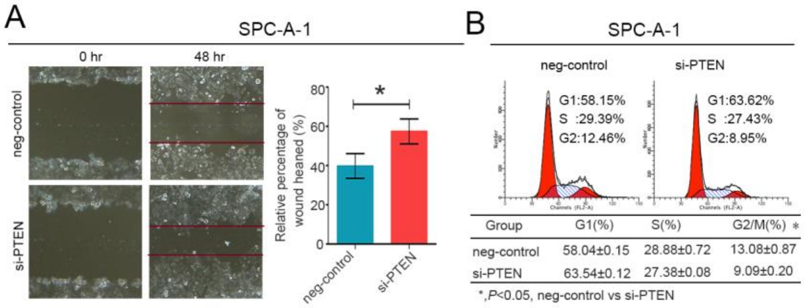

Group	G1(%)	S(%)	G2/M(%) *
neg-control	58.04±0.15	28.88±0.72	13.08±0.87
si-PTEN	63.54±0.12	27.38±0.08	9.09±0.20

*, *P*<0.05, neg-control vs si-PTEN

Supplementary Figure S2: (A) Would-healing assay for SPC-A-1 were performed with the negative control or si-PTEN. (B) Representative images and the table depict the results of cell cycle assays for SPC-A-1 were determined after transduction with the negative control or si-PTEN. The data are representative of three independent experiments. * *P*<0.05; ** *P*<0.01 by Student’s *t* test.

Supplementary Fig. S3

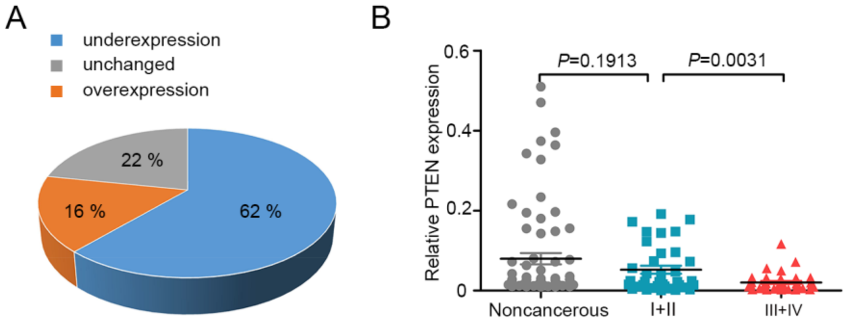

Supplementary Figure S3: Clinical validation of PTEN. **(A)** The relative expression of PTEN was performed by real-time PCR in 73 NSCLC samples and their corresponding noncancerous samples. (B) The expression of PTEN was detected in different clinical stages of NSCLC. The data are representative of three independent experiments. * *P*<0.05; ** *P*<0.01 by Student's *t* test.
